# Supplementary figures and images for: X-ray crystallographic and hydrogen deuterium exchange studies confirm alternate kinetic models for homolog insulin monomers
Source: PLoS One. 2025 Apr 21;20(4):e0319282. doi: 10.1371/journal.pone.0319282 (PMC12011231; doi:10.1371/journal.pone.0319282)

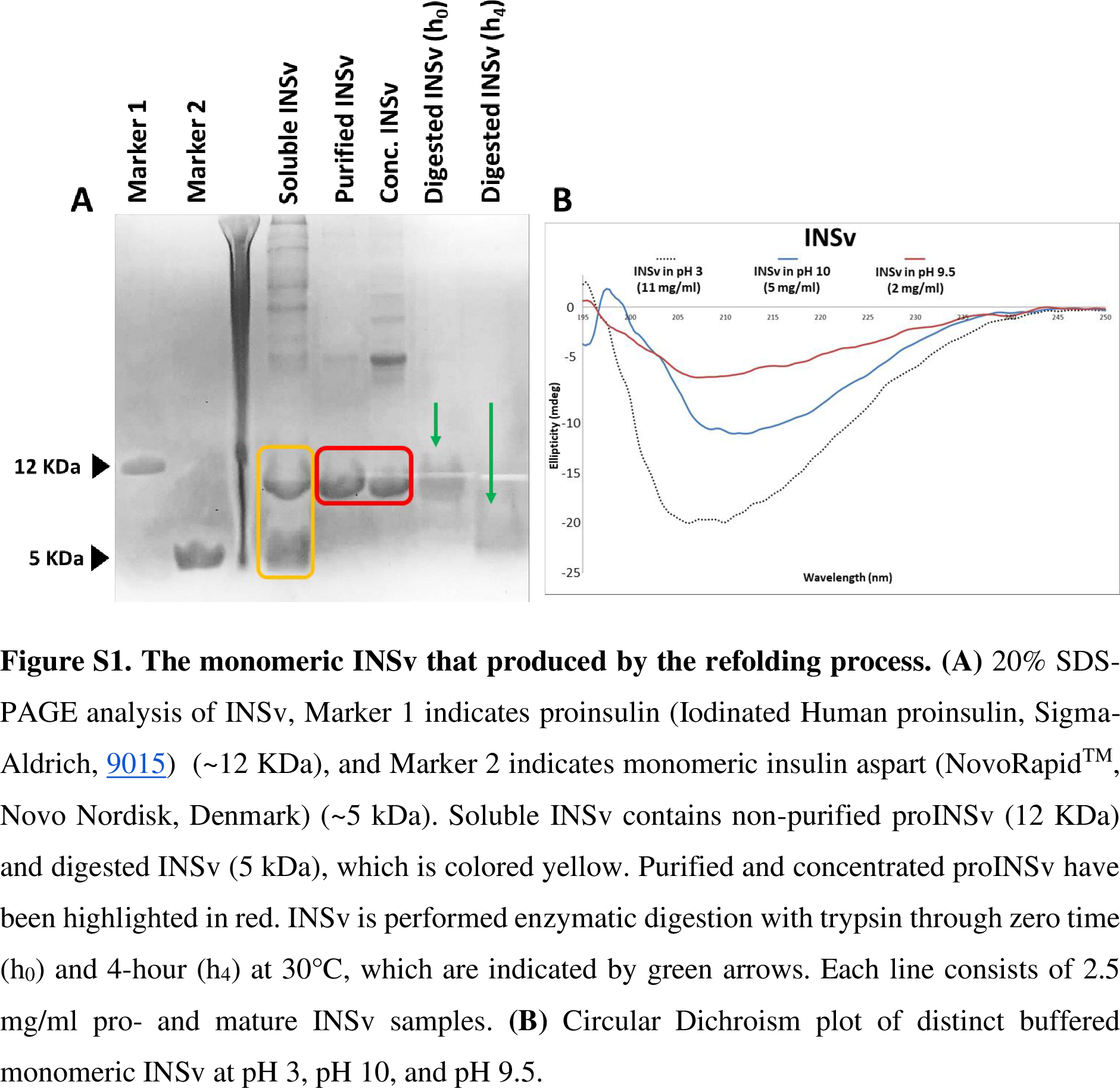

Supplement: S1 Fig — (A) 20% SDS-PAGE analysis of INSv, Marker 1 indicates proinsulin (Iodinated Human proinsulin, Sigma-Aldrich, 9015) (~12 KDa), and Marker 2 indicates monomeric insulin aspart (NovoRapidTM, Novo Nordisk, Denmark) (~5 kDa). Soluble INSv contains non-purified proINSv (12 KDa) and digested INSv (5 kDa), which is colored yellow. Purified and concentrated proINSv have been highlighted in red. INSv is performed enzymatic digestion with trypsin through zero time (h0) and 4-hour (h4) at 30°C, which are indicated by green arrows. Each line consists of 2.5 mg/ml pro- and mature INSv samples. (B) Circular Dichroism plot of distinct buffered monomeric INSv at pH 3, pH 10, and pH 9.5. (TIF) [file pone.0319282.s001.tif]

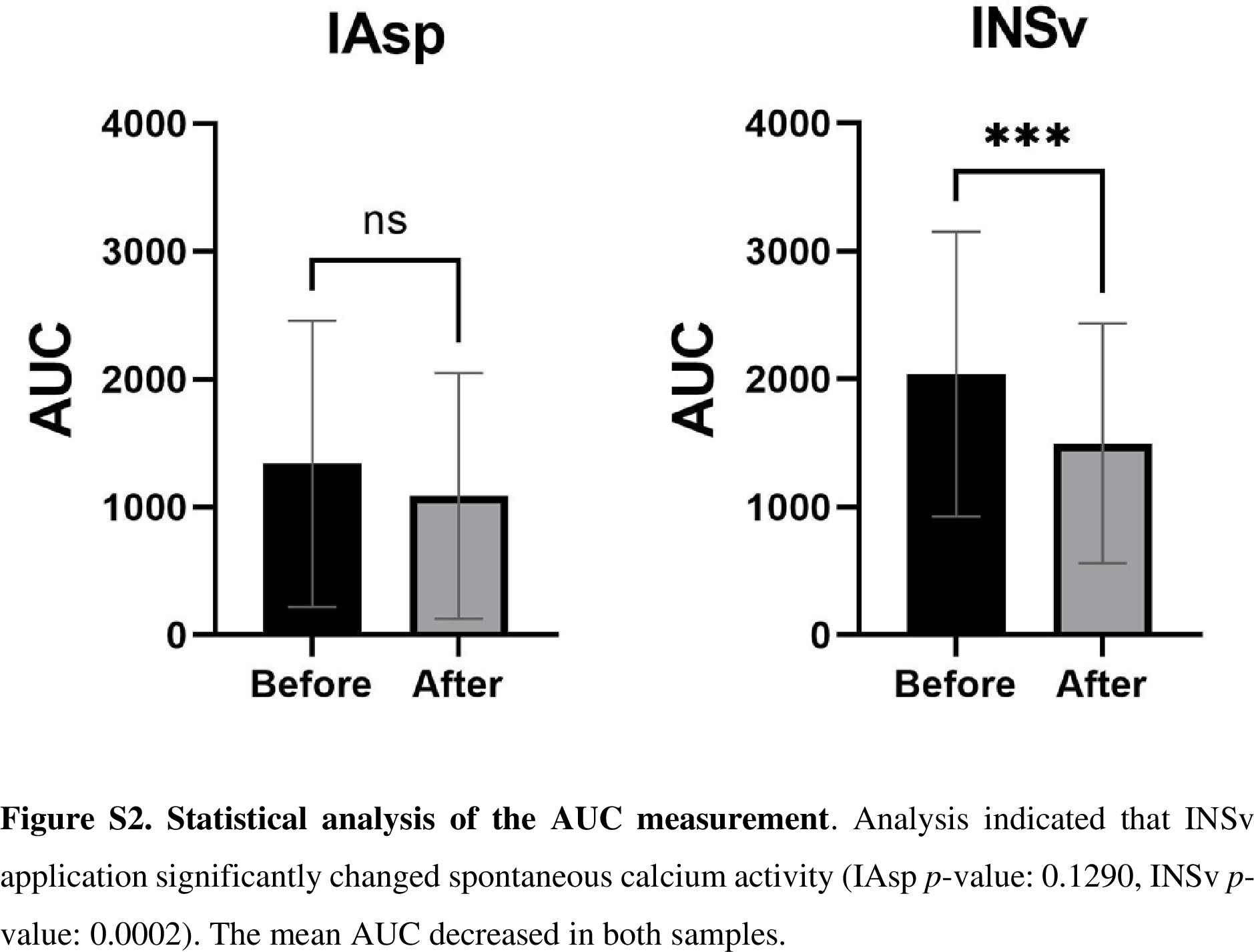

Supplement: S2 Fig — Analysis indicated that INSv application significantly changed spontaneous calcium activity (IAsp p-value: 0.1290, INSv p-value: 0.0002). The mean AUC decreased in both samples. (TIF) [file pone.0319282.s002.tif]

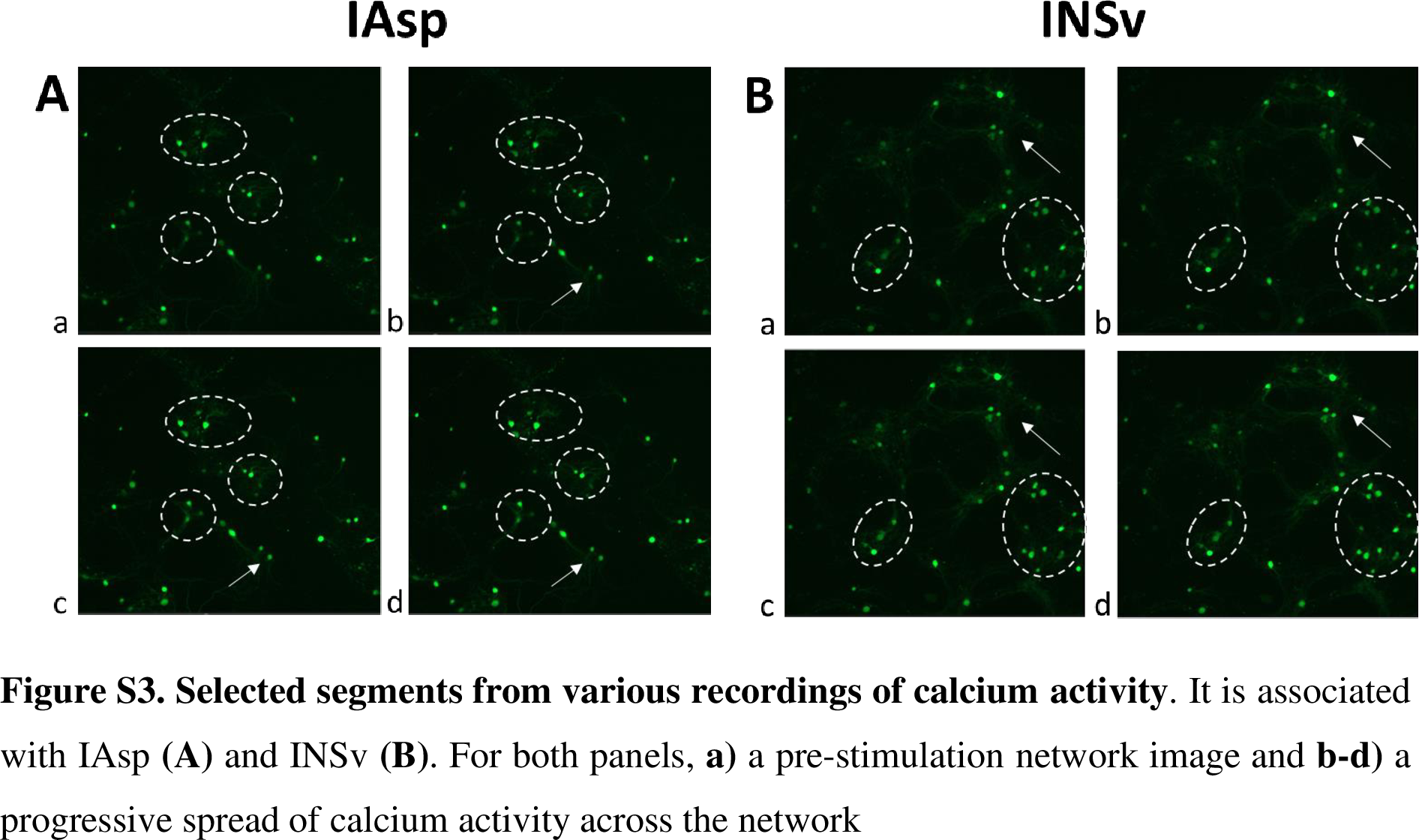

Supplement: S3 Fig — It is associated with IAsp (A) and INSv (B). For both panels, a) a pre-stimulation network image and b-d) a progressive spread of calcium activity across the network (TIF) [file pone.0319282.s003.tif]

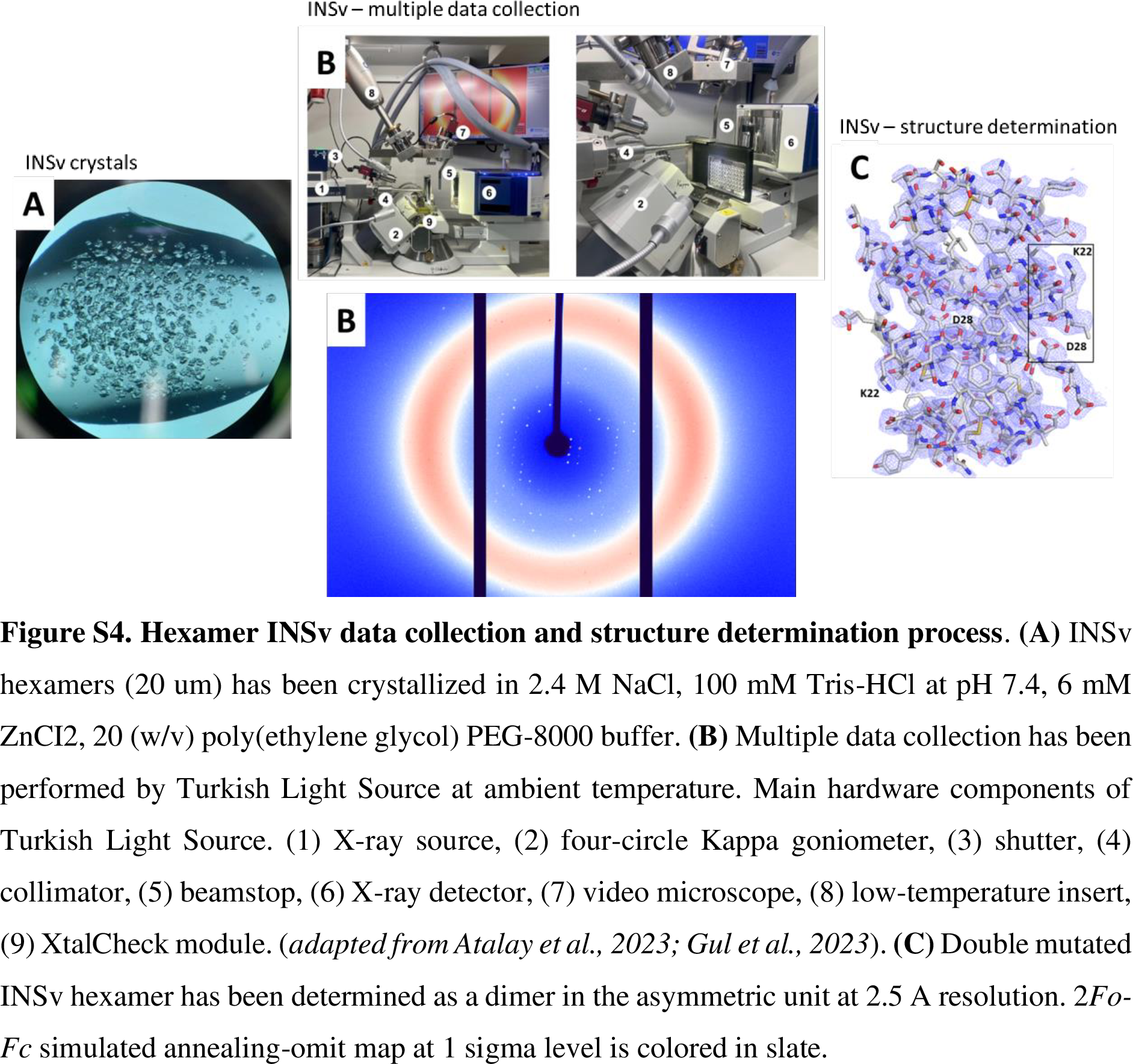

Supplement: S4 Fig — (A) INSv hexamers (20 um) has been crystallized in 2.4 M NaCl, 100 mM Tris-HCl at pH 7.4, 6 mM ZnCI2, 20 (w/v) poly(ethylene glycol) PEG-8000 buffer. (B) Multiple data collection has been performed by Turkish Light Source at ambient temperature. Main hardware components of Turkish Light Source. (1) X-ray source, (2) four-circle Kappa goniometer, (3) shutter, (4) collimator, (5) beamstop, (6) X-ray detector, (7) video microscope, (8) low-temperature insert, (9) XtalCheck module. (adapted from Atalay et al., 2023; Gul et al., 2023). (C) Double mutated INSv hexamer has been determined as a dimer in the asymmetric unit at 2.5 A resolution. 2Fo-Fc simulated annealing-omit map at 1 sigma level is colored in slate (TIF) [file pone.0319282.s004.tif]

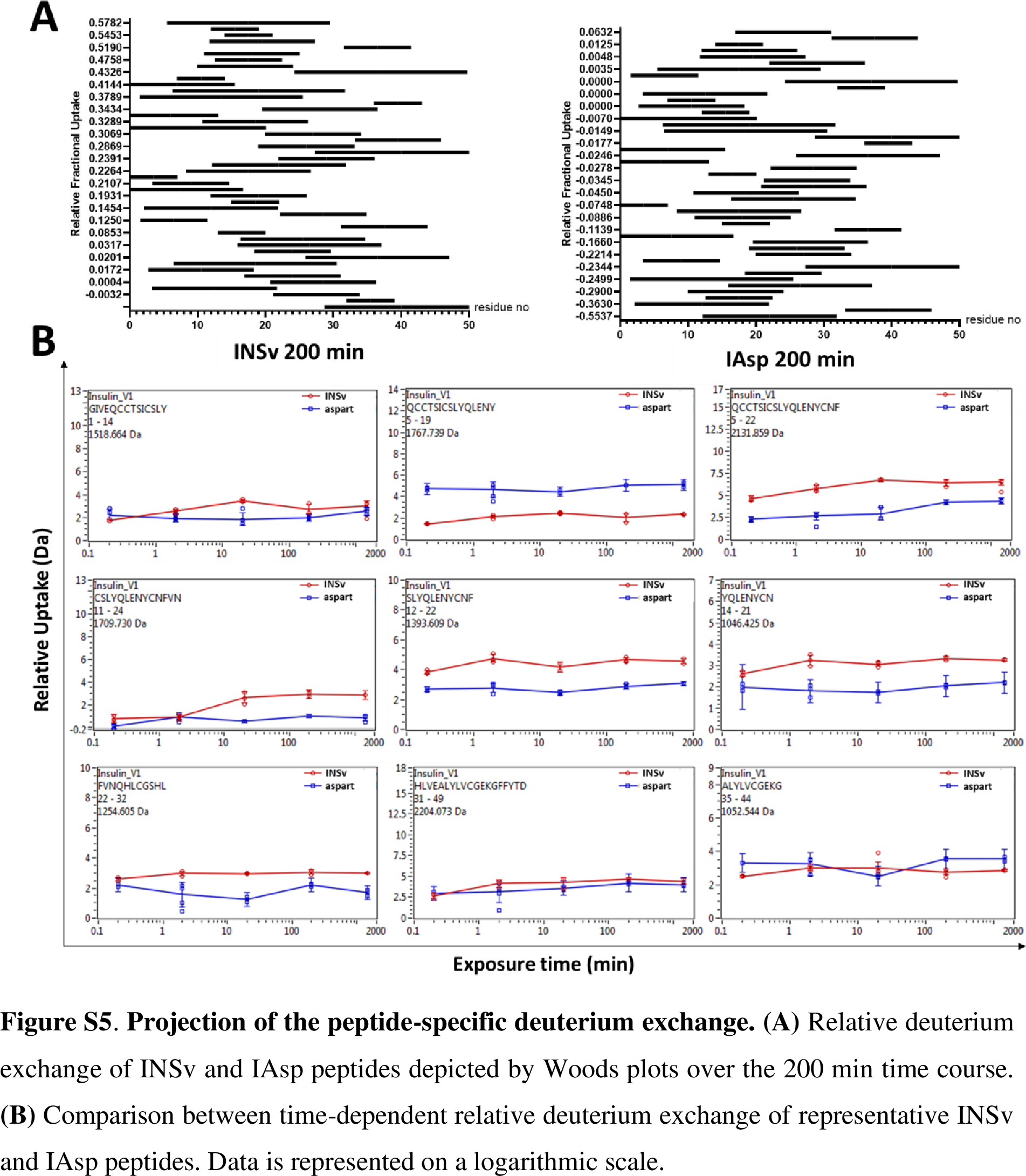

Supplement: S5 Fig — (A) Relative deuterium exchange of INSv and IAsp peptides depicted by Woods plots over the 200 min time course. (B) Comparison between time-dependent relative deuterium exchange of representative INSv and IAsp peptides. Data is represented on a logarithmic scale. (TIF) [file pone.0319282.s005.tif]

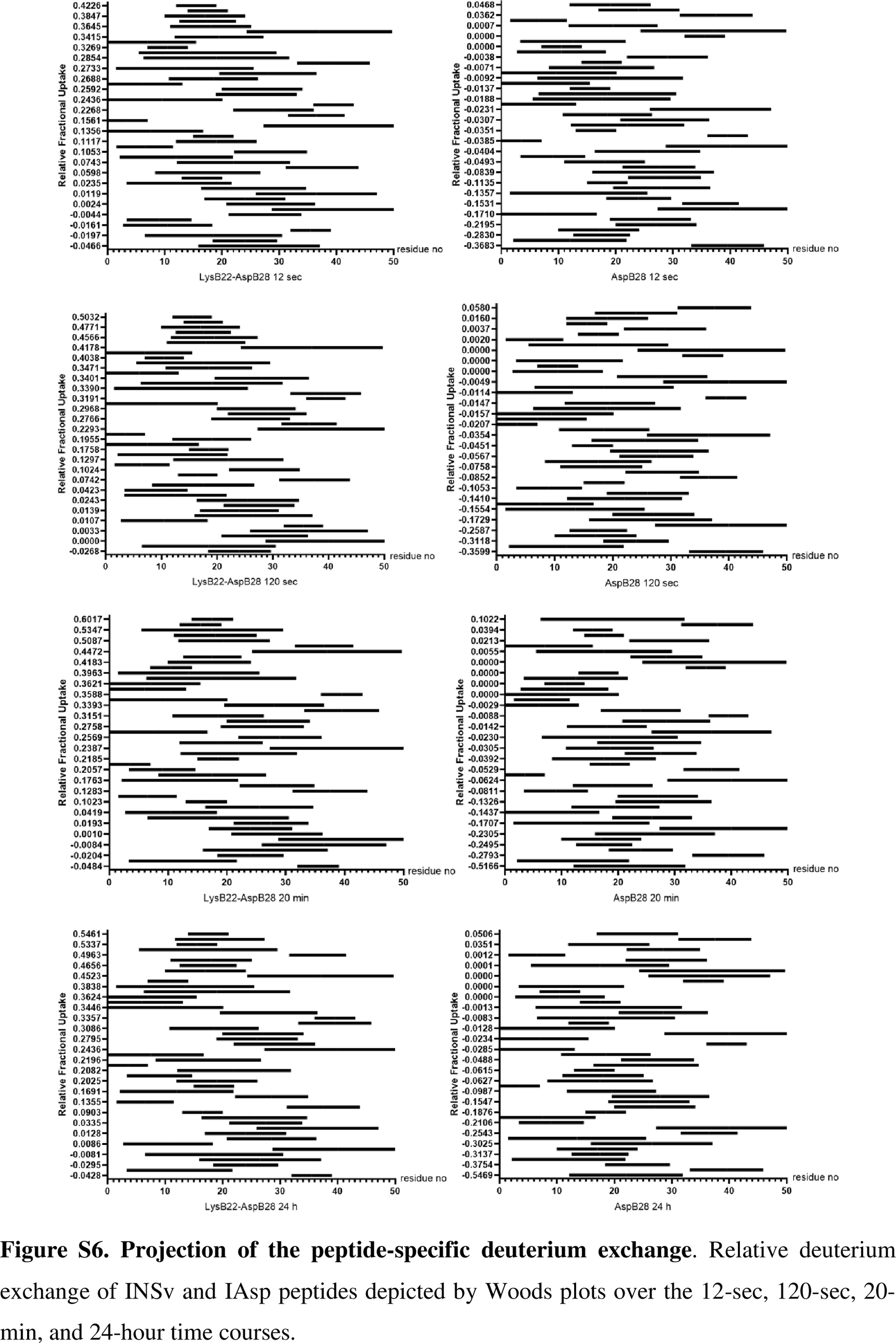

Supplement: S6 Fig — Relative deuterium exchange of INSv and IAsp peptides depicted by Woods plots over the 12-sec, 120-sec, 20-min, and 24-hour time courses (TIF) [file pone.0319282.s006.tif]

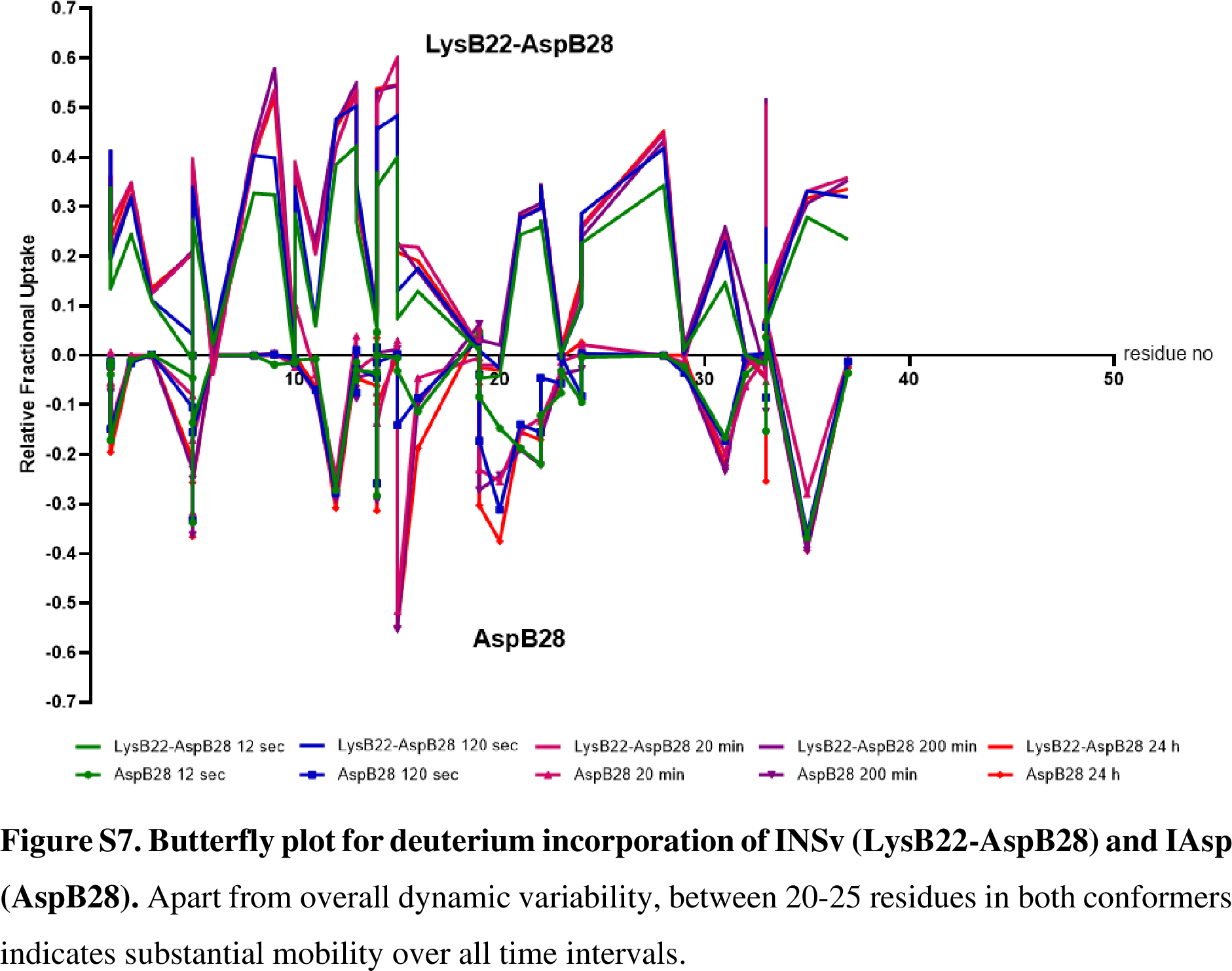

Supplement: S7 Fig — Apart from overall dynamic variability, between 20–25 residues in both conformers indicates substantial mobility over all time intervals (TIF) [file pone.0319282.s007.tif]

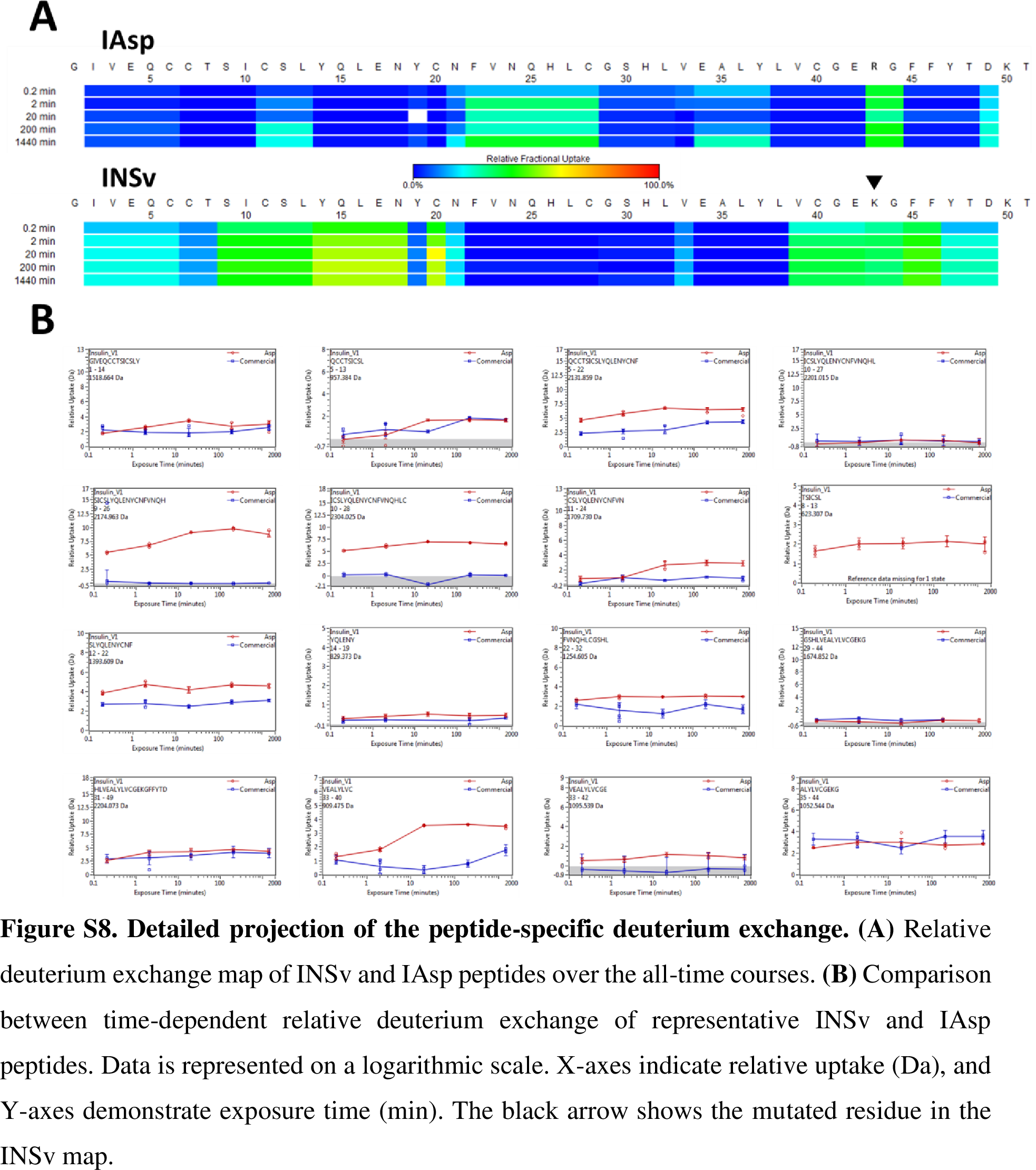

Supplement: S8 Fig — (A) Relative deuterium exchange map of INSv and IAsp peptides over the all-time courses. (B) Comparison between time-dependent relative deuterium exchange of representative INSv and IAsp peptides. Data is represented on a logarithmic scale. X-axes indicate relative uptake (Da), and Y-axes demonstrate exposure time (min). The black arrow shows the mutated residue in the INSv map. (TIF) [file pone.0319282.s008.tif]

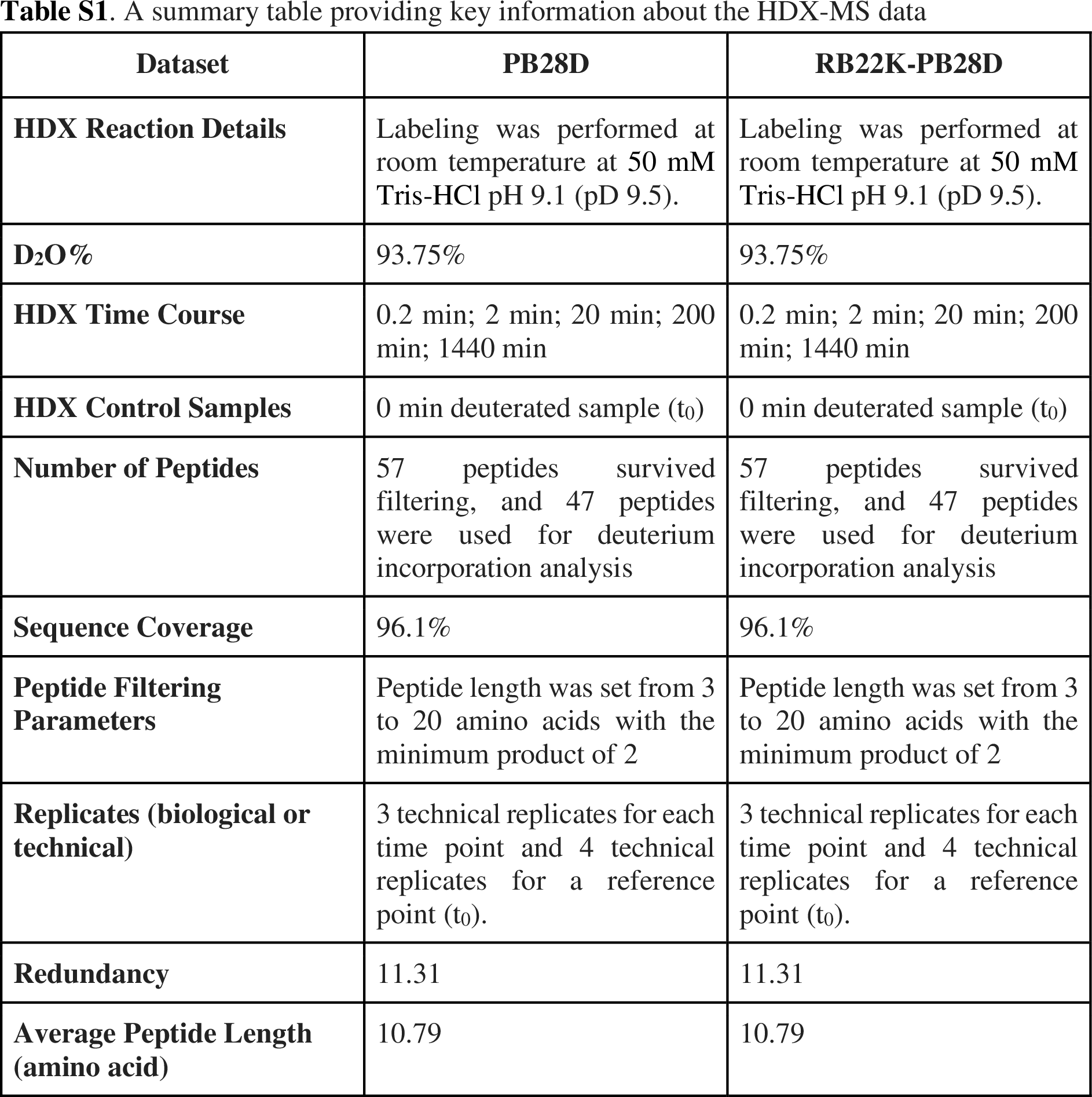

Supplement: S1 Table — (TIF) [file pone.0319282.s009.tif]
